# Pin1/YAP pathway mediates matrix stiffness‐induced epithelial–mesenchymal transition driving cervical cancer metastasis via a non‐Hippo mechanism

**DOI:** 10.1002/btm2.10375

**Published:** 2022-07-07

**Authors:** Long Yang, Jingwen Li, Guangchao Zang, Sijie Song, Zhengwen Sun, Xinyue Li, Yuanzhu Li, Zhenhong Xie, Guangyuan Zhang, Ni Gui, Shu Zhu, Tingting Chen, Yikui Cai, Yinping Zhao

**Affiliations:** ^1^ Laboratory of Tissue and Cell Biology Lab Teaching & Management Center, Chongqing Medical University Yuzhong District, Chongqing China

**Keywords:** epithelial–mesenchymal transition, extracellular matrix stiffness, PPIase non‐mitotic a‐interaction 1, yes‐associated protein

## Abstract

Cervical cancer metastasis is an important cause of death in cervical cancer. Previous studies have shown that epithelial–mesenchymal transition (EMT) of tumors promotes its invasive and metastatic capacity. Alterations in the extracellular matrix (ECM) and mechanical signaling are closely associated with cancer cell metastasis. However, it is unclear how matrix stiffness as an independent cue triggers EMT and promotes cervical cancer metastasis. Using collagen‐coated polyacrylamide hydrogel models and animal models, we investigated the effect of matrix stiffness on EMT and metastasis in cervical cancer. Our data showed that high matrix stiffness promotes EMT and migration of cervical cancer hela cell lines in vitro and in vivo. Notably, we found that matrix stiffness regulates yes‐associated protein (YAP) activity via PPIase non‐mitotic a‐interaction 1 (Pin1) with a non‐Hippo mechanism. These data indicate that matrix stiffness of the tumor microenvironment positively regulates EMT in cervical cancer through the Pin1/YAP pathway, and this study deepens our understanding of cervical cancer biomechanics and may provide new ideas for the treatment of cervical cancer.

## 1 INTRODUCTION

Cervical cancer metastasis is one of the important causes of death in cervical cancer.^1^, ^2^ The main ways of cervical cancer metastasis are interstitial infiltration, lymphatic metastasis, and hematogenous metastasis.^3^ During tumor metastasis, cancer cells often undergo epithelial–mesenchymal transition (EMT) to gain greater invasive and migratory capacity.^4^ EMT is a biological process in which epithelial cells are transformed into mesenchymal phenotype cells through a specific procedure, mainly manifested by decreased expression of epithelial markers such as E‐cadherin and enhanced expression of mesenchymal markers such as Vimentin, leading to enhanced cell migration.^5^ Recent studies have shown that EMT is not a binary process but a dynamic process in which cells in different stages of the process can coexist in the same tumor.^6^, ^7^, ^8^, ^9^ Importantly, cancer cells in a partial EMT state exhibit a more aggressive phenotype than those in a complete EMT state.^10^

Tumor metastasis is often closely related to the alteration of tumor microenvironment.^11^, ^12^, ^13^, ^14^ As an important component of the tumor microenvironment, extracellular matrix (ECM) stiffness not only maintains the three‐dimensional morphological structure of tumor tissue but also generates biochemical or biophysical signals that affect the biological functions of tumor cells.^15^, ^16^, ^17^ A study found that in cervical cancer, the involvement of cancer‐associated fibroblasts (CAF) in the remodeling of the ECM led to an increase in the stiffness of tumor tissue and promoted the progression of cervical cancer.^18^ In addition, higher matrix stiffness promotes cervical cancer cell proliferation and chemoresistance.^19^ However, past studies have focused on the role of various extracellular and intracellular biochemical signals in tumor progression,^20^, ^21^ with little knowledge on how matrix stiffness, simply as an independent cue, initiates EMT in cervical cancer cells.

Therefore, we aimed to investigate whether and how tissue mechanics affects EMT in cervical cancer. In this study, we elucidated the molecular mechanism by which matrix stiffness alone acts as an initiator to induce EMT in cervical carcinoma based on an in vitro collagen‐coated polyacrylamide (PA) hydrogel model with adjustable stiffness and an animal model. We verified the effect of matrix stiffness on EMT in cervical cancer and the role of yes‐associated protein (YAP) and PPIase non‐mitotic a‐interaction 1 (Pin1) in it. We further found that matrix stiffness regulates YAP activity through Pin1 in a non‐Hippo pathway. The present work highlights the important role of biomechanical signaling in triggering EMT and promoting invasive metastasis in cervical cancer, providing a promising idea for matrix stiffness therapy for the prevention of cervical cancer metastasis.

## 2 RESULTS

### 2.1 Matrix stiffness induces the occurrence of EMT in cervical cancer

**FIGURE 1:**
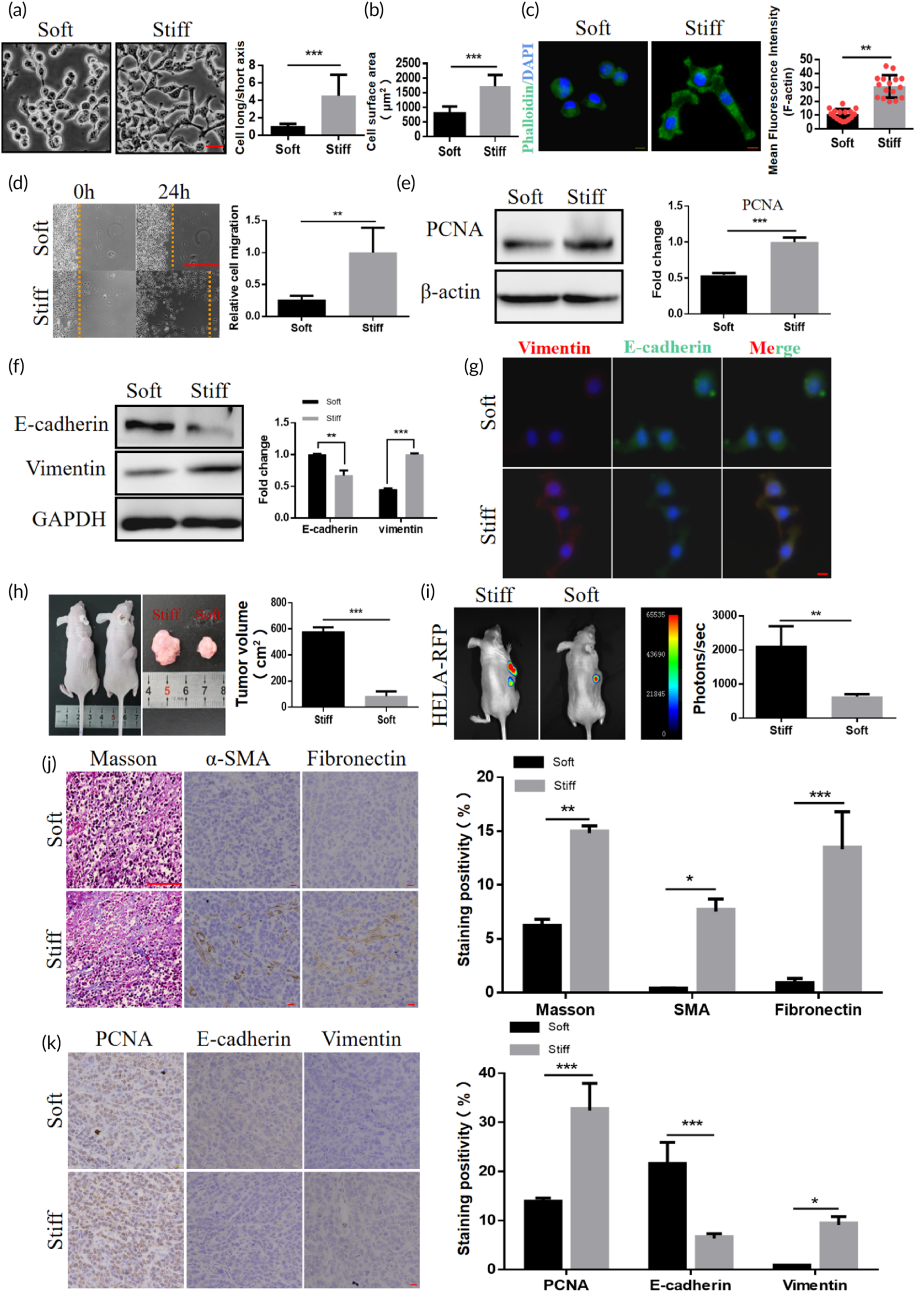
Matrix stiffness induces the occurrence of epithelial–mesenchymal transition (EMT) in cervical cancer. (a) Microscopy showing the typical morphology of hela cells cultured on soft and stiff matrix and quantification of cell long‐to‐short axis (***p < 0.001), scale bar: 50 μm. (b) Cell surface area was calculated by phase‐contrast analysis of cell images on soft and stiff matrix (***p < 0.001). (c) Immunofluorescence images of hela cells cultured on soft and stiff matrix and quantified for phalloidin (green) and 4',6‐diamidin0‐2‐phenylindole (DAPI) (blue) (**p < 0.01), scale bar: 10 μm. (d) Migration trajectory and quantification of hela cells cultured on soft or stiff matrix for 24 h (h) (**p < 0.01), scale bar: 100 μm. (e) Western blot analysis of proliferating cell nuclear antigen (PCNA) in hela cell, cultured on soft and stiff matrix showed expression and quantification. β‐Actin was used as a control (***p < 0.001). (f) Western blot analysis of E‐cadherin and Vimentin in hela cell, cultured on soft and stiff matrix showed expression and quantification, GAPDH was used as a control (***p < 0.001). (g) Immunofluorescence images of Vimentin (red), E‐cadherin (green) and DAPI (blue) in hela cells cultured on soft and stiff matrix, scale bar: 10 μm. (h) Hela‐RFP in situ xenografts were treated with dimethyl sulfoxide (DMSO) (stiff) or β‐aminopropionitrile (BAPN) (soft), and tumors were excised and measured in each group (*n* = 4, ***p < 0.001). (i) Representative bioluminescence images of mice (*n* = 4) in stiff and soft tumor groups after tumor implantation. Bar graphs show the quantification of total photon counts per group (*n* = 4, **p < 0.01). (j) Masson trichrome staining in stiff and soft tumor groups, scale bar: 100 μm. Immunohistochemical staining and quantification of α‐SMA and Fibronectin in stiff and soft tumor groups (*p < 0.05, **p < 0.01, ***p < 0.001), scale bar: 10 μm. (k) Immunohistochemical staining and quantification of PCNA, E‐cadherin, and Vimentin in stiff and soft tumor groups (*p < 0.05, ***p < 0.001), scale bar: 10 μm

Increased surface tension and stiffness of tumor cells have been reported to make them more biologically invasive.^17^ To investigate whether matrix stiffness can regulate the biological behavior of cervical cancer, we prepared a collagen‐coated polyacrylamide hydrogel (PA gel) system with Young's modulus of 1 and 20 kPa to simulate soft and stiff matrix in vitro.^22^ Under the microscope, significant differences in cell morphology were observed when hela cells were inoculated on a stiff and soft hydrogel. Cells on the soft matrix are round in shape, with round clusters of cells, whereas cells on the stiff matrix are elongated (Figure 1a) and significantly larger surface areas in response to stiff matrix (Figure 1b). In addition, F‐actin remodeling was observed in cells cultured on stiff matrix (Figure 1c), indicating that stiffness may influence cell motility. Compared with cells cultured on soft matrix, cells cultured on stiff matrix had a greater ability to migrate and proliferate (Figure 1d,e), indicating that matrix stiffness may influence malignant behavior of hela cells.

In the process of tumor metastasis, EMT often occur to enhance its invasions, among which the downregulation of epithelial marker E‐cadherin and the activation of mesenchymal marker Vimentin are regarded as the marker changes of EMT.^23^

Western blot and quantitative PCR (qPCR) results showed that E‐cadherin expression was reduced and Vimentin expression was increased in stiff matrix cells compared to soft matrix, and these results showed that matrix stiffness stimulation induced the occurrence of EMT in hela cells (Figure 1f and Figure S1). Interestingly, the induced EMT changes and associated signaling changes were reversed when cells initially cultured on stiff and re‐cultured on soft matrix (Figure S2A,B). To investigate the EMT status of cells undergoing matrix stiffness stimulation, we used immunofluorescence staining to observe the expression of E‐cadherin and Vimentin in cells cultured on soft and stiff matrix. The results showed a significant increase in Vimentin expression and decrease in E‐cadherin expression in cells cultured on stiff matrix compared to soft matrix, but cells on stiff matrix still expressed a small amount of E‐cadherin protein, which shows a presence of co‐expression of E‐cadherin and Vimentin in cells (Figure 1g), suggesting that cells undergoing matrix stiffness stimulation are experiencing a partial EMT state, which is consistent with the performance described in previous studies.^24^, ^25^

In order to further study the effect of matrix stiffness to the hela cells in vivo, we established a cell‐derived xenograft model and applied β‐aminopropionitrile (BAPN), a classical drug that reduces the mechanical properties of ECM, to reduce the stiffness of tumors in vivo.^26^, ^27^ To correspond to the previous section, we refer to the BAPN treatment group as the soft group and the dimethyl sulfoxide (DMSO) control group as the stiff group. The size of xenograft tumor was significantly reduced in the soft group compared to the stiff group (Figure 1h). Furthermore, the soft group also had a lower tumor burden compared to the stiff group (Figure 1i). Masson staining and immunohistochemical staining with α‐smooth muscle actin (α‐SMA) and fibronectin showed that tumor fibrosis was significantly downregulated in the soft group compared with the stiff group (Figure 1j). In addition, tumor in soft group showed lower proliferating cell nuclear antigen (PCNA) expression than stiff group (Figure 1k), indicating that reduced matrix stiffness inhibits tumor proliferation in vivo. Immunohistochemical staining of E‐cadherin and Vimentin showed less Vimentin expression and more E‐cadherin expression in soft group than in stiff group, indicating that reduced matrix stiffness can inhibit tumor EMT in vivo (Figure 1k). Taken together, in vitro and in vivo data indicate that matrix stiffness modulates the EMT of cervical cancer.

### 2.2 Matrix stiffness modulates EMT in cervical cancer by promoting YAP nuclear localization

YAP is widely present in vivo and functions as a sensor and mediator of mechanical signals indicative of the cellular microenvironment.^28^ As a typical mechanism of YAP regulation, the phosphorylation cascade reaction of the Hippo pathway has been identified.^29^ The Hippo pathway phosphorylates YAP through large tumor suppressor (LATS), causing it to stay in the cytoplasm to inhibit its activity, while dephosphorylated YAP enters the nucleus with the transcriptional enhancer subdomain (TEAD), regulating the transcription of specific genes thereby promoting cell proliferation and survival.^30^, ^31^

**FIGURE 2:**
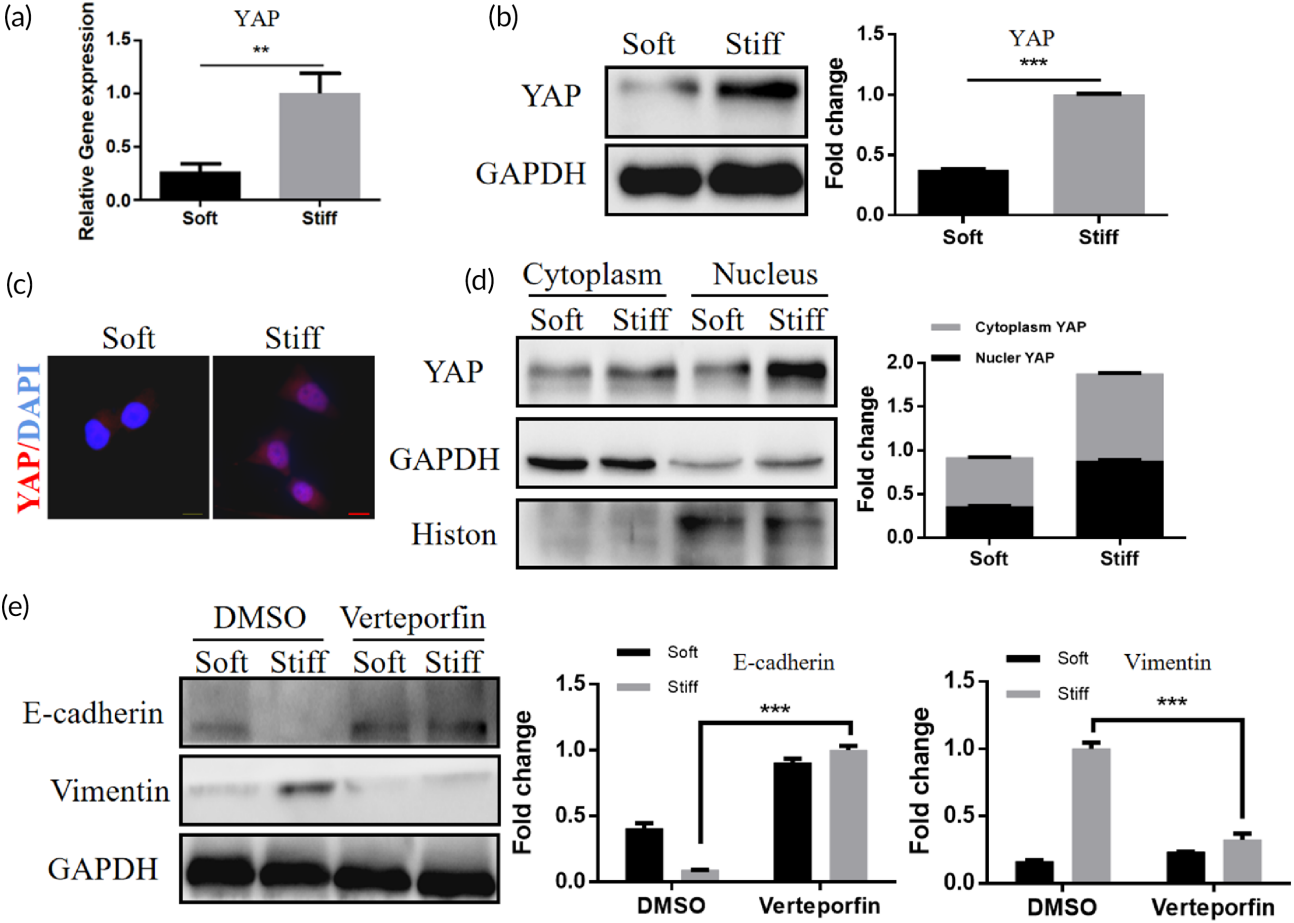
Matrix stiffness modulates epithelial–mesenchymal transition (EMT) in cervical cancer by promoting YAP nuclear localization. (a) Relative mRNA expression of YAP in hela cells cultured on soft and stiff matrix measured by qPCR (**p < 0.01). (b) Western blot analysis of YAP in hela cell, cultured on soft and stiff matrix showed expression and quantification, GAPDH was used as a control (***p < 0.001). (c) Immunofluorescence images of YAP (red) and DAPI (blue) in hela cells cultured on soft and stiff matrix, scale bar: 10 μm. (d) Western blot analysis and quantification of YAP in nuclear and cytoplasm, GAPDH was used as a cytoplasm control and histone as a nuclear control. (e) Western blot analysis of E‐cadherin and Vimentin in hela cell treated with DMSO and Verteporfin, GAPDH was used as a control (***p < 0.001).

As YAP activation in many tumors can lead to EMT,^32^ we investigated whether matrix stiffness can induce EMT in hela cells by YAP. The expression of YAP in hela cells incubated on stiff and soft hydrogels were analyzed by western blot and qPCR. Compared with soft matrix cultured cells, the stiff matrix cultured cells had higher YAP mRNA expression (Figure 2a) and protein expression (Figure 2b). Immunostaining showed a significant increase in YAP nuclear localization of hela cells cultured on a stiff matrix (Figure 2c). Western blotting of nuclear and cytoplasmic proteins revealed that stiff matrix cultured cells showed more obvious YAP nuclear aggregation (Figure 2d). To investigate the role of YAP in EMT of hela cells, we used the YAP–TEAD complex inhibitor verteporfin to inhibit the activation of YAP,^33^ and the western blotting results showed that verteporfin treatment reversed the promotion of cellular EMT by matrix stiffness (Figure 2e). These results indicate that matrix stiffness can induce cellular EMT through activation of YAP and that verteporfin inhibition of YAP activity can reverse its pathological process.

### 2.3 Matrix stiffness modulates EMT in cervical cancer by modulating Pin1 activity

**FIGURE 3:**
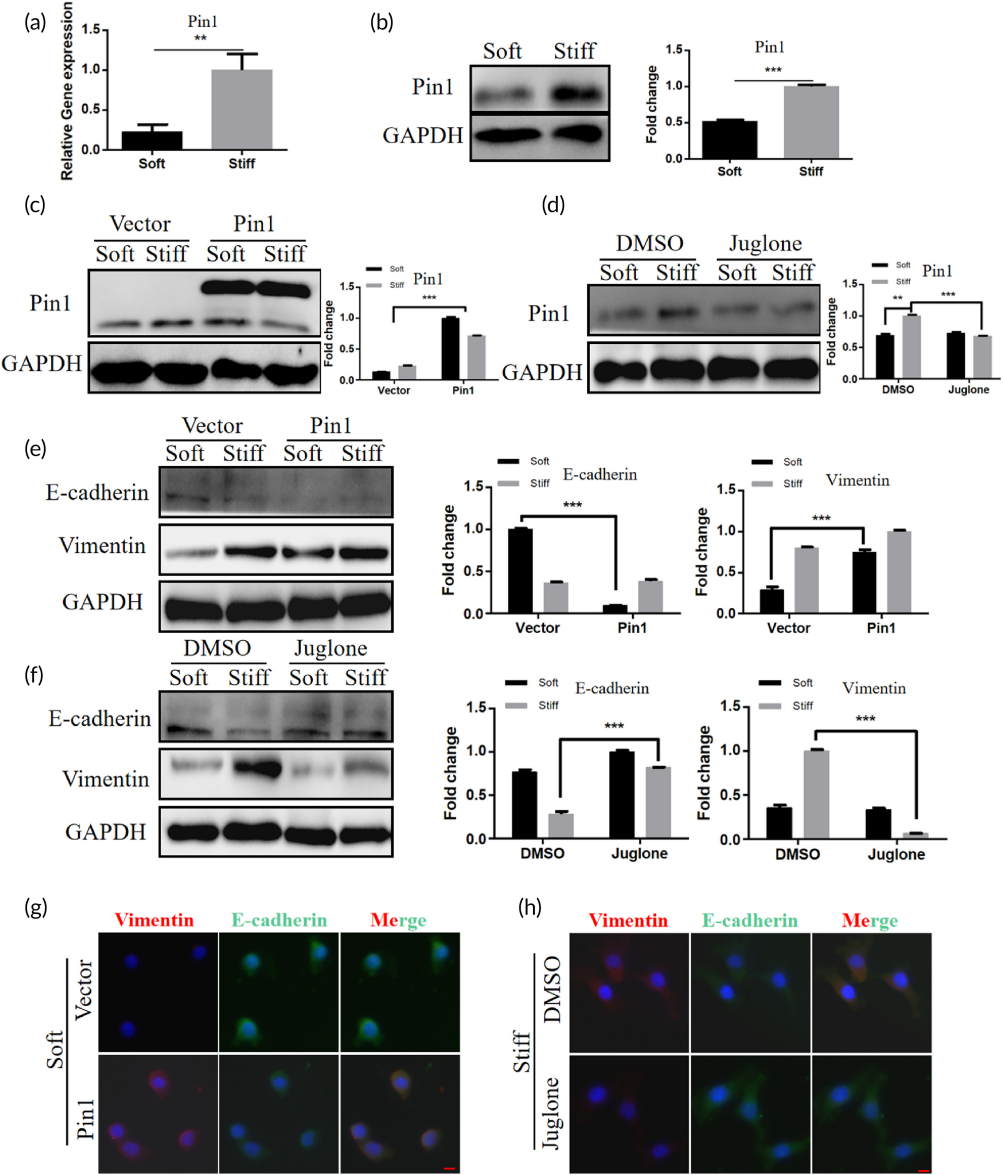
Matrix stiffness modulates EMT in cervical cancer by modulating Pin1 activity. (a) Relative mRNA expression of Pin1 in hela cells cultured on soft and stiff matrix measured by qPCR (**p < 0.01). (b) Western blot analysis of Pin1 in hela cell, cultured on soft and stiff matrix showed expression and quantification, GAPDH was used as a control (***p < 0.001). (c) Western blot analysis of Pin1 in hela cell treated with empty vector and Pin1 plasmid showed expression and quantification, GAPDH was used as a control (***p < 0.001). (d) Western blot analysis of Pin1 in hela cell treated with DMSO and Juglone showed expression and quantification, GAPDH was used as a control (***p < 0.001). (e) Western blot analysis of E‐cadherin and Vimentin in hela cell treated with empty vector and Pin1 plasmid showed expression and quantification, GAPDH was used as a control (***p < 0.001). (f) Western blot analysis of E‐cadherin and Vimentin in hela cell treated with DMSO and Juglone showed expression and quantification, GAPDH was used as a control (***p < 0.001). (g,h) Immunofluorescence images of Vimentin (red), E‐cadherin (green) and DAPI (blue) in hela cells overexpressing Pin1 cultured on soft matrix and hela cells inhibited Pin1 cultured on stiff matrix, scale bar: 10 μm

Pin1 is a member of the peptidyl prolyl isomerase (PPIase) family^34^ that specifically binds proteins containing phosphorylated Ser/Thr‐Pro motifs that alters the function of phosphorylated proteins to regulate signal transduction after phosphorylation, which plays an important role in cancer and neuropathy.^35^, ^36^ Inhibition of Pin1 has been reported to significantly reduce the proliferation and metastasis of cervical cancer cells, and it may serve as a new therapeutic target for cervical cancer.^37^ To investigate whether Pin1 is involved in the regulation of EMT in hela cells by matrix stiffness, we examined the expression of Pin1 in hela cells inoculated on stiff and soft hydrogels using western blotting and qPCR. The results showed that mRNA expression (Figure 3a) and protein expression (Figure 3b) of Pin1 were higher in cells on stiff matrix than on soft matrix. To determine the role of Pin1 in the regulation of EMT in cervical cancer by matrix stiffness, we upregulated Pin1 expression in hela cells using an expression plasmid and inhibited Pin1 expression in hela cells using the Pin1 inhibitor Juglone^38^, ^39^ and verified its effect using western blotting (Figure 3c,d). Western blotting results showed that overexpression of Pin1 increased the level of Vimentin expression and decreased the level of E‐cadherin expression in hela cells cultured especially on soft matrix (Figure 3e). In contrast, inhibition of Pin1 expression reduced Vimentin expression and restored E‐cadherin expression, especially in cells cultured on stiff matrix (Figure 3f). In addition, when Pin1 expression was downregulated, the proliferation of hela cells cultured under stiff matrix was reduced more significantly (Figure S3). Immunofluorescence staining showed that cells cultured on soft matrix showed co‐expression of E‐cadherin and Vimentin after overexpression of Pin1, which indicated that Pin1 was able to induce partial EMT on soft matrix (Figure 3g), which consistent with the published data.^40^ In contrast, inhibition of Pin1 reversed the stiff matrix‐induced EMT state (Figure 3h).

### 2.4 Matrix stiffness modulates YAP nuclear translocation via Pin1 in a non‐Hippo pathway

**FIGURE 4:**
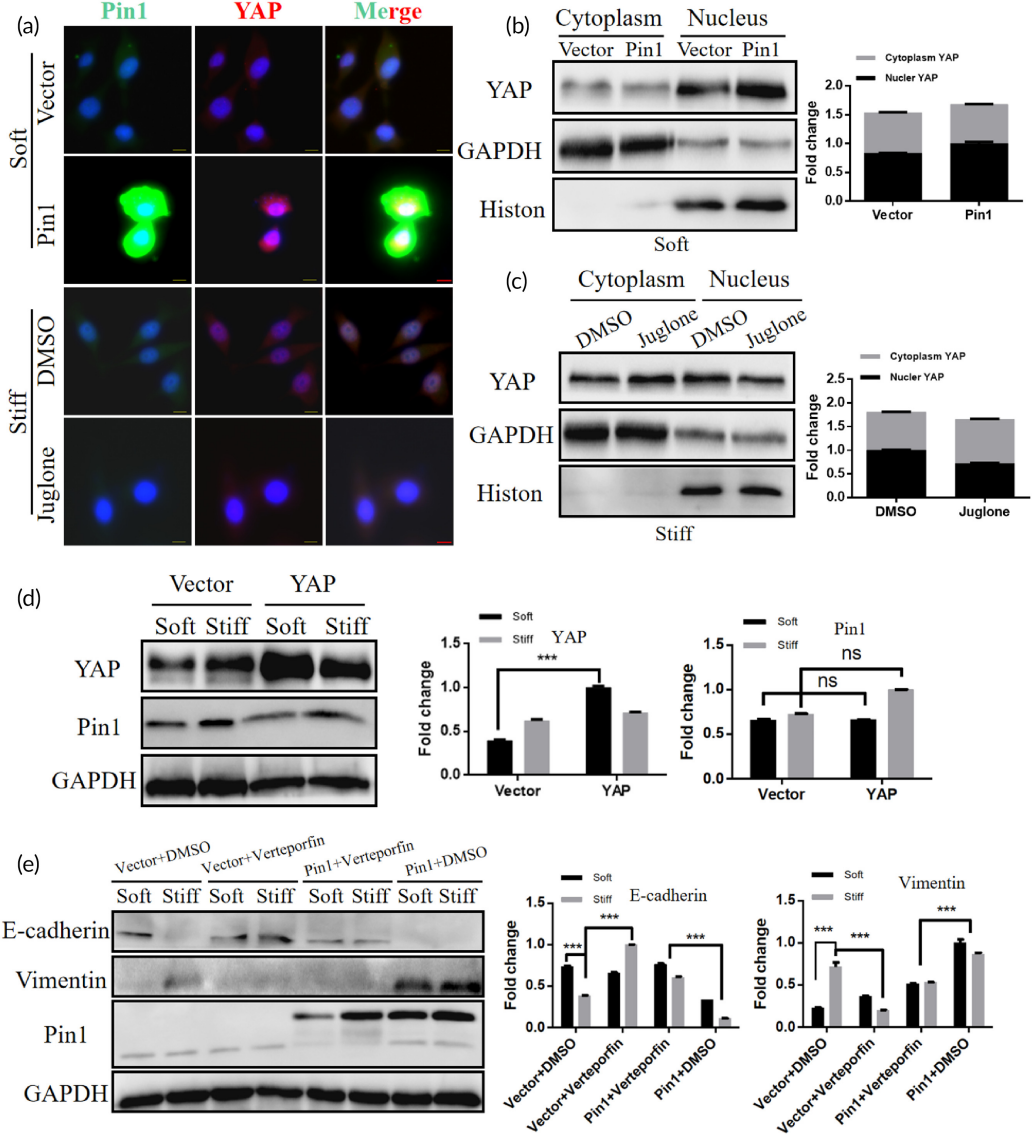
Matrix stiffness can promote YAP nuclear translocation via Pin1. (a) Immunofluorescence images of YAP (red), Pin1 (green) and DAPI (blue) in hela cells overexpressing Pin1 cultured on soft matrix and hela cells inhibited Pin1 cultured on stiff matrix, scale bar: 10 μm. (b) Westernblot analysis and quantification of YAP in the nucleus and cytoplasm of Pin1 overexpressing cells on soft matrix, GAPDH was used as cytoplasmic control and histone as nuclear control. (c) Westernblot analysis and quantification of YAP in the nucleus and cytoplasm of hela cells inhibited Pin1 cultured on stiff matrix, GAPDH was used as a cytoplasmic control and histone as a nuclear control. (d) Western blot analysis of Pin1 and YAP in hela cell treated with empty vector and YAP plasmid showed expression and quantification, GAPDH was used as control (ns means no significance, ***p < 0.001). (e) Western blot analysis of E‐cadherin and Vimentin in hela cell treated with empty vector, Pin1 plasmid, DMSO and Verteporfin showed expression and quantification, GAPDH was used as control (***p < 0.001).

The current study shows that Pin1, as a positive regulator of YAP, can increase the activity and stability of YAP.^41^ In addition, Pin1 interacts with STK3 (MST2), an upstream regulator of the Hippo pathway, to regulate STK3 activity, which in turn promotes YAP entry into the nucleus and exerts biological effects.^42^ Thus, we investigated whether Pin1 mediated the activation of matrix stiffness regulated YAP. First, we overexpressed Pin1 in soft matrix cultured cells using expression plasmids and inhibited Pin1 in stiff matrix cultured cells using Juglone. By immunostaining and Westernblot analysis of nuclear and cytoplasmic, we found that Pin1 overexpression promoted YAP nuclear localization in soft matrix cultured cells, while Pin1 inhibition decreased YAP nuclear localization in stiff matrix cultured cells (Figure 4a–c). To resolve the upstream–downstream relationship between YAP and Pin1, we performed reverse experiments. First, we used a YAP expression plasmid to overexpress YAP in cells cultured on soft and stiff matrix and found that overexpression of YAP did not alter the expression of Pin1 (Figure 4d). We also treated hela cells transfected with the Pin1 expression plasmid using the YAP inhibitor verteporfin. The results show that verteporfin reverses the cellular EMT alterations promoted by Pin1 overexpression (Figure 4e). Taken together, our results indicate that matrix stiffness can promote YAP nuclear translocation via Pin1.

**FIGURE 5:**
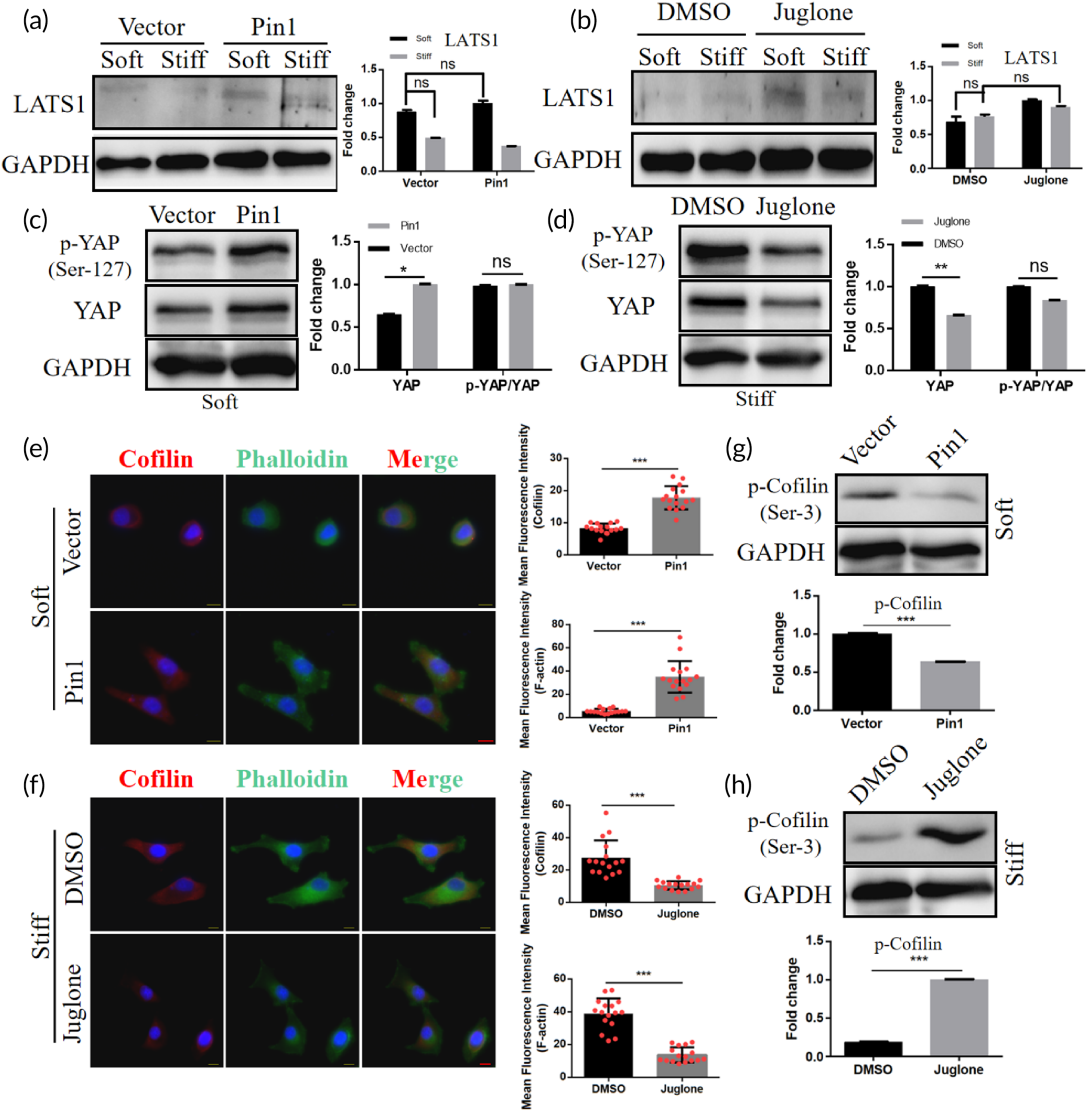
Matrix stiffness regulates YAP nuclear translocation via Pin1 in a non‐Hippo pathway. (a) Western blot analysis of LATS1 in hela cell treated with empty vector and Pin1 plasmid showed expression and quantification. GAPDH was used as control (“ns” means no significance). (b) Western blot analysis of LATS1 in hela cell treated with DMSO and Juglone showed expression and quantification. GAPDH was used as a control (“ns” means no significance). (c) Western blot analysis and quantification of YAP, p‐YAP in cells overexpressing Pin1 on soft matrix. GAPDH and YAP was used as a control (“ns” means no significance, *p < 0.05). (d) Western blot analysis and quantification of YAP, p‐YAP in cells inhibiting Pin1 cultured on stiff matrix. GAPDH and YAP was used as a control (“ns” means no significance, **p < 0.01). (e) Immunofluorescence images and quantification of Cofilin (red), phalloidin (green) and DAPI (blue) in hela cells overexpressing Pin1 cultured on soft matrix, scale bar: 10 μm (***p < 0.001). (f) Immunofluorescence images and quantification of Cofilin (red), phalloidin (green) and DAPI (blue) in hela cells inhibited Pin1 cultured on stiff matrix, scale bar: 10 μm (***p < 0.001). (g) Western blot analysis and quantification of Cofilin in cells overexpressing Pin1 on soft matrix. GAPDH was used as a control (***p < 0.001). (h) Western blot analysis and quantification of Cofilin on cells inhibiting Pin1 on stiff matrix. GAPDH was used as a control (***p < 0.001).

The phosphorylation cascade of Hippo pathway modulates the phosphorylation of YAP is currently the classical theory to explain the cause of YAP nuclear translocation.^43^ Among them, LATS1, as one of the cores of Hippo pathway, plays a key role in the phosphorylation of YAP by Hippo pathway.^44^ To investigate whether Pin1 affects YAP via Hippo pathway kinase under matrix stiffness stimulation, we first performed western blotting assays on LATS1 protein and found that neither Pin1 overexpression nor inhibition treatment altered LATS1 expression (Figure 5a,b). Then, we overexpressed Pin1 in cells cultured on soft and stiff matrix using expression plasmids and inhibited Pin1 in cells cultured on stiff matrix using Juglone and analyzed intracellular YAP and phosphorylated YAP by western blot. The results showed that Pin1 overexpression increased the expression of YAP protein in cells on soft matrix (Figure 5c), while Pin1 inhibition decreased the expression of YAP protein in cells on stiff matrix (Figure 5d). However, the ratio of phosphorylated YAP to total YAP did not change in either overexpressed or inhibited cells, indicating that the regulation of YAP by Pin1 was not through changing the phosphorylation of YAP. Thus, we reasoned that Hippo pathway kinase may not be an indispensable mediator for Pin1 to regulate YAP activity.

Current studies indicate that in addition to the classical Hippo pathway, there are non‐Hippo pathways such as α‐catenin^45^ and AMOT^46^ that can regulate YAP activity.^45^ For example, a recent study^47^ indicates that remodeling of F‐actin can directly regulate YAP activity through non‐Hippo pathway pathways. In the preceding Figures 1c and 3b, it can be seen that there is a positive correlation between the increase in Pin1 expression under matrix stiffness stimulation and F‐actin remodeling, indicating whether Pin1 may directly regulate YAP nuclear translocation by regulating F‐actin remodeling.

To test this claim, we overexpressed Pin1 in hela cells cultured on soft matrix and inhibited Pin1 in hela cells cultured on stiff matrix and then observed immunofluorescence staining for F‐actin and Cofilin, a key factor regulating F‐actin remodeling. After overexpression of Pin1, cells on soft matrix showed increased Cofilin expression and F‐actin content, and the cell morphology was more similar to that of shuttle shape (Figure 5e). In contrast, after Pin1 inhibition, cells on the stiff matrix showed reduced Cofilin expression, reduced F‐actin content, and more rounded cell morphology (Figure 5f). Meanwhile, western blotting results of phosphorylated Cofilin showed that cells on soft matrix showed decreased expression of phosphorylated Cofilin after overexpression of Pin1 (Figure 5g), indicating increased Cofilin activity. In contrast, cells on stiff matrix showed increased expression of phosphorylated Cofilin after Pin1 inhibition (Figure 5h), indicating diminished Cofilin activity. Interestingly, after we treated hela cells with Latrunculin B (F‐actin polymerization inhibitor) and Jasplakinolide (F‐actin polymerization and stabilization inducer), the results showed that F‐actin polymerization increased Pin1 expression, while inhibition of F‐actin polymerization decreased Pin1 expression (Figure S4A,b), indicating a possible positive feedback relationship between F‐actin and Pin1. Taken together, we reveal that Pin1 regulates YAP nuclear translocation through F‐actin remodeling independent of Hippo pathway kinase.

### 2.5 Pin1 modulates EMT in cervical cancer in vivo

To further investigate the role of Pin1 in hela cell EMT in vivo, we used the Pin1 inhibitor Juglone to treat xenograft tumor.

**FIGURE 6:**
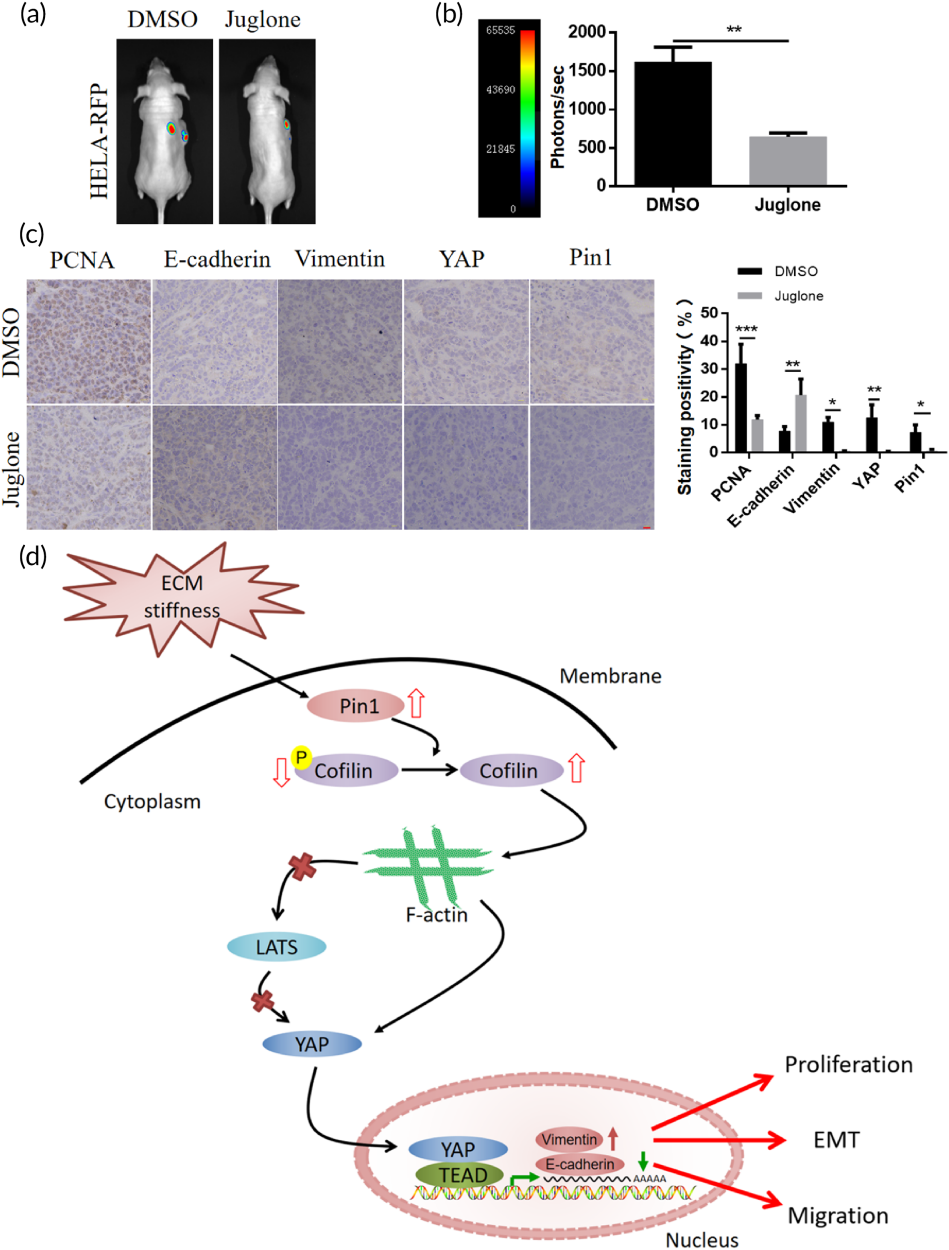
Pin1 modulates EMT in cervical cancer in vivo. (a) Representative bioluminescence images of xenograft tumor treated with DMSO and Juglone (*n* = 4). (b) Bar graphs show the quantification of total photon counts per group (*n* = 4, **p < 0.01). (**c**) Immunohistochemical staining and quantification of PCNA, E‐cadherin, Vimentin, YAP and Pin1 in DMSO and Juglone treated tumors (*p < 0.05, **p < 0.01, ***p < 0.001), scale bar: 10 μm. (d) Schematic model of molecular signaling of Pin1/YAP pathway mediates matrix stiffness‐induced epithelial–mesenchymal transition driving cervical cancer metastasis via a non‐Hippo mechanism

The results showed a reduced tumor burden in Juglone‐treated tumor (Figure 6a,b) and immunohistochemistry showed a reduction in PCNA expression of Juglone‐treated tumor (Figure 6c), indicating that Pin1 inhibition inhibits tumor proliferation in vivo. Immunohistochemical staining showed that E‐cadherin expression was more in the Juglone‐treated tumor than in the DMSO treated tumor, and Vimentin expression was less than in the DMSO‐treated tumor (Figure 6c), indicating that downregulation of Pin1 expression inhibits EMT in cervical cancer in vitro. In addition, we found that inhibition of Pin1 in vivo can lead to downregulation of YAP expression (Figure 6c), indicating that Pin1 regulate EMT in hela cell via YAP pathway in vivo.

## 3 DISCUSSION

In this study, we demonstrated that matrix stiffness induces the occurrence of EMT in cervical cancer. Notably, we found that the activity of Pin1 was able to change with the stimulation of matrix stiffness. More importantly, we found that matrix stiffness regulates YAP nuclear translocation via Pin1 in a non‐Hippo pathway thus promote EMT in cervical cancer. This study provides evidence that targeting ECM stiffness can hinder EMT in cervical cancer and indicates that Pin1 and YAP may be a promising target for treatment against cervical cancer.

The EMT program is modulated by the network of multiple pro‐EMT factors such as TWIST1. Recent studies showed that matrix stiffness drives EMT and tumor metastasis through a TWIST mechanotransduction pathway.^24^, ^48^ They also noticed matrix rigidity triggers YAP nuclear localization in breast cancer. However, there are distinct differences between the mechanotransduction pathways of YAP/TAZ and TWIST1. TWIST1 are only sensitive in matrix stiffness, while YAP/TAZ also make response to the cellular polarity, shape and adherens junction. Besides, they treated MCF10A cells with leptomycin B, a nuclear export inhibitor, and found that YAP accumulated into the nucleus on compliant matrix whereas TWIST1 not. These researches mainly focus on molecular pathway of TWIST1 signaling axis, the YAP/TAZ mechanotransduction pathway remains to be elucidated. The present study shows that Pin1 activity varied with matrix stiffness and was able to regulate F‐actin remodeling, probably because Pin1 expression increased Cofilin activity, thus regulating F‐actin remodeling. More importantly, we found that matrix stiffness regulates YAP nuclear translocation via Pin1 in a non‐Hippo pathway thus promoting EMT in cervical cancer. We found that Pin1 regulates YAP nuclear translocation in a nonphosphorylation‐dependent manner, the main reason is quite likely the cytosolic pore opening caused by skeletal alterations which in turn leads to direct entry of YAP into the nucleus.^49^, ^50^ But all of the altered import and export rates caused by nuclear pore opening experimental phenomena have not been explained by a reasonable model,^45^ this remains to be investigated.

Multiple recent reports showed EMT and matrix stiffness have reciprocal crosstalk on each other. A previous study showed that lung cancer cells that have undergone EMT can activate the FAK/SRC axis by altering the collagen composition of their surrounding microenvironment, leading to invasion and metastasis.^51^ Their study also revealed that miR‐200 and ZEB1 can affect the migration ability of mesenchymal lung cancer cells in vitro through direct regulation of LOX proteins. A recent study also demonstrated that cancer cells can maintain the stability of the mesenchymal state of the cell through the ZEB1‐LOXL2‐ECM circuit and make the return to the epithelial phenotype more difficult, which has important implications for the distant metastasis of cancer cells.^52^ These results suggest that LOX may serve as a potential clinical therapeutic target. Gibbons's team^51^ used BAPN drugs to inhibit LOX enzyme activity, and transwell migration and collagen invasion assays found that lung cancer cell migration was inhibited in vitro, while *in* mice vivo experiments did not show significant change in tumor size. In our study, inhibition of ECM stiffness using BAPN significantly impeded the ability of metastasis in cervical cancer, and these results are consistent with several studies confirming the effectiveness of chemical LOX inhibitors in vivo.^27^, ^53^, ^54^, ^55^, ^56^ Their results are not consistent with ours, which may be due to an off‐target effect. They also explored the consideration of whether there is a link between LOX and YAP activity, but this remains to be further investigated.

It is worth to note not all tumor types are highly sensitive to the ECM stiffness stimulation.^15^, ^57^ The crosstalk of ECM stiffness and tumor cells has been well studied in adenocarcinoma such as breast cancer in contrast to squamous cell carcinoma. Interestingly, there might be differences exist between carcinoma types. Mammary epithelial cells initially cultured on stiff and re‐cultured on soft matrix, exhibit active migration behaviors after re‐cultured on soft matrix.^58^ This phenomenon is defined as cellular memory, meaning that past exposure to different values of matrix stiffness affects the behavior of cells.^58^, ^59^, ^60^, ^61^ However, as an example of oral squamous cell carcinoma (ORCC), it exhibits a dual phenotype in which cells do not appear to exhibit cellular memory when cells initially cultured on stiff and re‐cultured on soft matrix, while those re‐cultured on a stiff matrix appear to be primed for migration.^62^ Our results are consistent with theirs in which the EMT and associated signaling changes induced by stiff matrix were reversed when hela cell initially cultured on stiff and re‐cultured on soft matrix, which shows cells did not appear to exhibit mechanical memory. Therefore, it would be important to further investigate the mechanical memory of cells and the role of Pin1/YAP in it.

In summary, the present study demonstrated from in vivo and in vitro experiments that matrix stiffness promotes EMT in cervical cancer, and the Pin1/YAP pathway plays an important role in this (Figure 6d). This may provide new ideas for the design of clinical drug targets.

## 4 MATERIALS AND METHODS

### 4.1 Cell culture

The human cervical cancer cell lines hela and hela‐RFP were cultured in dulbecco's modified eagle medium (DMEM) medium (Gibco) supplemented with 10% fetal bovine serum (FBS) (Biological Industries) and 100 units/ml of penicillin/streptomycin (Hyclone). All cells were cultured at 37°C and 5% CO_2_ (Thermo fisher scientific).

### 4.2 Antibodies and reagents

Complete information on all antibodies and reagents used in this study are provided in Table S1 and S2.

### 4.3 Transfection

pcDNA3.1‐Flag‐Pin1 was purchased from Kepilon Biotechnology (Chongqing, China). pcDNA3.1‐HA‐YAP and pcDNA3.1 are friendly gifts from Prof. Nan Lu (Chongqing Medical University). Plasmids were transfected using transfection reagent Lipofectamine 3000 (Thermo Fisher scientific), and experiments were performed according to the manufacturer's instructions.

### 4.4 Immunofluorescence

IF was performed according to a method described previous.^63^ The cells were fixed with 4% paraformaldehyde (Leagene) for 30 min at room temperature (RT), permeabilized with 0.1% TritonX‐100 (Solarbio) for 20 min at room temperature and then closed with 5% BSA (Solarbio) for 20 min. Primary antibodies were incubated overnight at 4°C, followed by incubation in DyLight‐coupled secondary antibodies for 1 h. Cytoskeleton staining was then incubated with FITC‐labeled phalloidin (Yisheng Biotechnology) for 1 h. Nucleus were stained with DAPI (Solarbio). Immunofluorescence microscopy was observed for staining (Nikon Ti2).

### 4.5 Preparation of PA hydrogels

Preparation of PA hydrogels was performed according to a method described previous.^22^ PA hydrogels with soft and stiff stiffness were prepared by adjusting the relative concentrations of acrylamide (Aladdin) and bisacrylamide (Aladdin). First, the acrylamide and bisacrylamide fractions were mixed proportionally according to the desired stiffness. The solution was sterilized by filtration through a 0.22 μm filter. The dissolved oxygen in the solution is removed by vacuum. Then 3‐aminopropyltrimethoxysilane (APES; Sangon) was added to the surface of the glass coverslip for 5 min and rinsed completely with distilled water. Subsequently, N,N,N',N'‐Tetramethylethylenediamine (TEMED) (Aladdin) and 10% ammonium persulfate (Aladdin) were added to a mixed solution of acrylamide and bisacrylamide for polymerization. Prior to the polymerization of the solution, it was transferred to a glass slide and covered with a glass coverslip. After the polymerization was completed in about 15 min, the hydrogel was removed from the slides and coverslips for the next step of the experiment. To promote cell adhesion, a protein coating was applied to the surface of the hydrogel, sufosuccinimidyl‐6‐(4′‐azido‐2′‐nitrophenylamino)‐hexanoate (sulfo‐SANPAH; ProteoChem) was applied to the surface of the hydrogels and irradiated at 365 nm of UV light for 20 min. The synthetic gels were then washed with 50 mM 2‐[4‐(2‐hydroxyethyl)piperazin‐1‐yl]ethanesulfonic acid (HEPES) buffer (Sangon), incubated with 10 μg/ml rat tail collagen I (Solarbio) solution at 4°C overnight, and sterilized under UV light for 20 min.

### 4.6 Animal studies

Cell‐derived xenograft model was performed according to a method described previous.^27^ Female BALB/c‐nu mice (5 weeks old) were purchased from Enswell Biotechnology Ltd (Chongqing, China). All animal experiments were approved by the Animal Ethics Committee of Chongqing Medical University, and all procedures were in accordance with the National Institute of Health “China Animal Research Guidelines” (“Guidelines for the Protection and Use of Laboratory Animals”). All mice were housed in pathogen‐free conditions at 22°C in the Animal Research Center of Chongqing Medical University, with a 12‐h light/dark cycle and free access to water and food. We established a cervical cancer xenograft tumor model by using 200 μl PBS lysed 5 × 10^6^ cells per nude mouse injected subcutaneously into the axilla. Two weeks after cell injection, mice were randomly divided into control, BAPN‐ and Juglone‐treated groups (*n* = 16). For BAPN treatment, BAPN (50 mg/kg) was injected intraperitoneally every 2 days for 4 weeks. When Juglone treatment was performed, intraperitoneal injection of Juglone (3 mg/kg) was used every 2 days for 4 weeks. DMSO was used as a control. After 4 weeks of injection, mice were anesthetized with 5% chloral hydrate and imaged with a fluorescence imaging system (OI600 Touch, BIO‐OI). After mice were operated, tumors were excised and frozen or fixed in paraformaldehyde in preparation for the next experiments.

### 4.7 Total and nuclear‐cytosol protein extraction and Western blot analysis

Total proteins were extracted from cells using RIPA lysis buffer containing protease inhibitors (Beyotime). Nuclear and cytoplasmic proteins were extracted from cells using the Nucleocytoplasmic Extraction Kit (Beyotime) according to the manufacturer's instructions. WB analysis was carried out as previously reported.^64^ For westernblot analysis, primary antibody designation is shown in Table S1.

### 4.8 Quantitative PCR analysis

Total RNA was extracted from treated cells using Trizol (Absin) and reverse transcribed into cDNA using the PrimeScriptRT kit (Takara). Experiments were performed using faststart essential DNA green master (Roche) according to the reagent vendor's instructions. mRNA expression of E‐cadherin, Vimentin, YAP, Pin1 was determined using the 2^−ΔΔ^CT method. GAPDH was used as a control. The primer sequences were as follows:

YAP (forward: 5'‐TCGGCAGGCAATACGGAATA‐3′; reverse: 5′‐CATGCTGAGGCCACTGTCTGT‐3′);

Pin1 (forward: 5′‐TCGGGAGAGGAGGACTTTG‐3′; reverse: 5′‐GGAGGATGATGTGGATGCC‐3′);

E‐cadherin (forward: 5′‐ATTTTTCCCTCGACACCCGAT‐3′; reverse: 5′‐TCCCAGGCGTAGACCAAGA‐3′);

Vimentin (forward: 5′‐TGCCGTTGAAGCTGCTAACTA‐3′; reverse: 5′‐CCAGAGGGAGTGAATCCAGATTA‐3′);

GAPDH (forward: 5′‐TGACTCTACCCACGGCAAGTTCAA‐3′; reverse: 5′‐ACGACATACTCAGCACCAGCATCA‐3′).

### 4.9 Masson staining and immunochemistry

Tumor tissues were fixed in 4% paraformaldehyde (Leagene), embedded in paraffin, and then sectioned (5 μm). For Masson's trichrome staining, a standard protocol was used to assess fibrosis.^65^ For immunohistochemical analysis,^65^ the primary antibody designation is shown in Table S1. These images were taken with a microscope (Nikon Ti2).

### 4.10 Cell migration assay

First, the cells were inoculated in a six‐well plate placed on hydrogel slides, partially covered by sterile slides so as to form a clear “edge” without physically harming the cell monolayer. Then incubated for 24 h to form a fused monolayer, after which the covered slides were carefully removed. Cells were rinsed twice with PBS and photographed under a microscope (Nikon Ti2). Subsequently, fresh DMEM containing 2% fetal bovine serum was added to six‐well plate culture dishes and incubated in an incubator for 24 h. At last, the well plates were removed and photographed under the microscope to observe cell migration. The experiment was repeated three times under each treatment condition. Quantitative assessment was performed using ImageJ software. The performed method was carried out as previously reported.^66^

### 4.11 Statistical analysis

All experiments were repeated thrice; numerical data are expressed as mean ± standard error (SEM), and statistical significance (defined as *p* < 0.05) was determined using the two‐sample *t*‐test of GraphPadPrism6.

## AUTHOR CONTRIBUTIONS

**Long Yang:** Conceptualization (equal); data curation (lead); investigation (lead); writing – original draft (lead); writing – review and editing (lead). **Jingwen Li:** Data curation (supporting); investigation (supporting); writing – review and editing (supporting). **Guangchao Zang:** Data curation (supporting); investigation (supporting); writing – original draft (supporting). **Sijie Song:** Investigation (supporting). **Zhengwen Sun:** Investigation (supporting). **Xinyue Li:** Data curation (supporting); writing – review and editing (supporting). **Yuanzhu Li:** Data curation (supporting); writing – review and editing (supporting). **Zhenhong Xie:** Investigation (supporting). **Guangyuan Zhang:** Writing – original draft (supporting). **Ni Gui:** Investigation (supporting). **Shu Zhu:** Writing – review and editing (supporting). **Tingting Chen:** Writing – original draft (supporting). **Yikui Cai:** Investigation (supporting). **Yinping Zhao:** Conceptualization (lead); data curation (supporting); formal analysis (lead); project administration (lead); writing – original draft (supporting).

## Supporting information


**Appendix S1** Supporting information

## Data Availability

The data that support the findings of this study are available from the corresponding author upon reasonable request.
