# Supplementary material for: Pin1/YAP pathway mediates matrix stiffness‐induced epithelial–mesenchymal transition driving cervical cancer metastasis via a non‐Hippo mechanism
Source: Bioeng Transl Med. 2022 Jul 7;8(1):e10375. doi: 10.1002/btm2.10375 (PMC9842039; doi:10.1002/btm2.10375)
Supplement: Supplementary file 1 — Appendix S1 Supporting information [file BTM2-8-e10375-s001.docx]

**Supplementary Table 1.** Details on the antibodies used in the present study

| **Antibodies** | **Source** | **Dilution** | **Catalogue number** | **Manufacturer** |
| --- | --- | --- | --- | --- |
| Anti-YAP antibody | Mouse | WB: 1:1500  IF:1:200 | sc-101199 | [Santa Cruz](https://www.baidu.com/link?url=6XMGAn-9DaQcAlppOj6dYBnHbq09ttisc_dYC-7dFLu&wd=&eqid=c6579b700002fb720000000662382bea) |
| Anti-Cofilin 1 antibody | Mouse | IF:1:200 | sc-53934 | Santa Cruz |
| Anti-Fibronectin antibody | Rabbit | IHC:1:200 | ab268020 | Abcam |
| Anti-α-Smooth Muscle Actin antibody | Rabbit | IHC: 1:200 | 19245S | [Cell Signaling Technology](http://www.baidu.com/link?url=LEdue1PRYc7VdMS4zR6PPi1QJdF_7JBOrswcUbfs9JlmvQlflV8lBPf_EvXDyQBT) |
| Anti-Phospho-YAP (Ser127) antibody | Rabbit | WB: 1:1500 | 13008S | [Cell Signaling Technology](http://www.baidu.com/link?url=LEdue1PRYc7VdMS4zR6PPi1QJdF_7JBOrswcUbfs9JlmvQlflV8lBPf_EvXDyQBT) |
| Anti-Pin1 antibody | Rabbit | WB: 1:1500 | 3722S | [Cell Signaling Technology](http://www.baidu.com/link?url=LEdue1PRYc7VdMS4zR6PPi1QJdF_7JBOrswcUbfs9JlmvQlflV8lBPf_EvXDyQBT) |
| Anti-Pin1 antibody | Rabbit | IF: 1:200  IHC:1:200 | 10495-1-AP | [Proteintech](https://www.baidu.com/link?url=mqXL0JCE06yhgCXA8oRyqazqrlpgr-_mxtm-hnTS0q7&wd=&eqid=f40bddd0000145c60000000662383f86) |
| Anti-PCNA antibody | Rabbit | WB: 1:1500  IHC:1:200 | 10205-2-AP | Proteintech |
| Anti-Beta Actin antibody | Rabbit | WB: 1:1500 | 20536-1-AP | Proteintech |
| Anti-E-cadherin antibody | Rabbit | WB: 1:1500 | bs-1519R | Bioss |
| Anti-Vimentin antibody | Rabbit | WB: 1:1500 | bs-0756R | Bioss |
| Anti-E-cadherin antibody | Rabbit | IF: 1:200 | 20874-1-AP | [Proteintech](https://www.baidu.com/link?url=mqXL0JCE06yhgCXA8oRyqazqrlpgr-_mxtm-hnTS0q7&wd=&eqid=f40bddd0000145c60000000662383f86) |
| Anti-Vimentin antibody | Mouse | IF: 1:200 | 60330-1-Ig | Proteintech |
| Anti-LATS1 antibody | Rabbit | WB: 1:1500 | bs-2904R | Bioss |
| Anti-YAP antibody | Rabbit | IHC: 1:200 | ET1608-30 | Huabio |
| Anti-Vimentin antibody | Rabbit | IHC:1:200 | PB9359 | Boster |
| Anti-E-cadherin antibody | Rabbit | IHC: 1:200 | PB9561 | Boster |
| Anti-GAPDH antibody | Rabbit | WB: 1:2500 | [D110016](https://www.sangon.com/productDetail?productInfo.code=D110016) | Sangon |
| HRP-conjugated Goat Anti-Rabbit IgG | Goat | WB: 1:5,000  IHC:1:1000 | D110058 | Sangon |
| HRP-conjugated Goat Anti-Mouse IgG | Goat | WB: 1:5,000 | D110087 | Sangon |
| Dylight 594 Goat Anti-Mouse IgG | Goat | IF:1:200 | A23410 | Abbkine |
| Dylight 488, Goat Anti-Rabbit IgG | Goat | IF:1:200 | A23220 | Abbkine |

IHC, immunohistochemistry; WB, western blotting. IF: immunofluorescence

**Supplementary Table 2.** Details on the reagents used in the present study

| **Reagents** | **Source** | **Identifier** |
| --- | --- | --- |
| Juglone | Sigma-Aldrich | H47003 |
| β-Aminopropionitrile | MCE | HY-Y1750 |
| Verteporfin | MCE | HY-B0146 |
| Latrunculin B | Cayman | 76343-94-7 |
| Jasplakinolide | Cayman | 102396-24-7 |
| Sulfo-SANPAH | ProteoChem | [102568-43-4](https://china.guidechem.com/322976/) |
| Collagen Type Ⅰ | Solarbio | C8062 |
| DMSO | Sangon | A503039 |

**Supplementary Figure 1.** Relative mRNA expression of E-cadherin and Vimentin in hela cells cultured on soft and stiff matrix measured by quantitative PCR (***P*<0.01, ****P*<0.001).


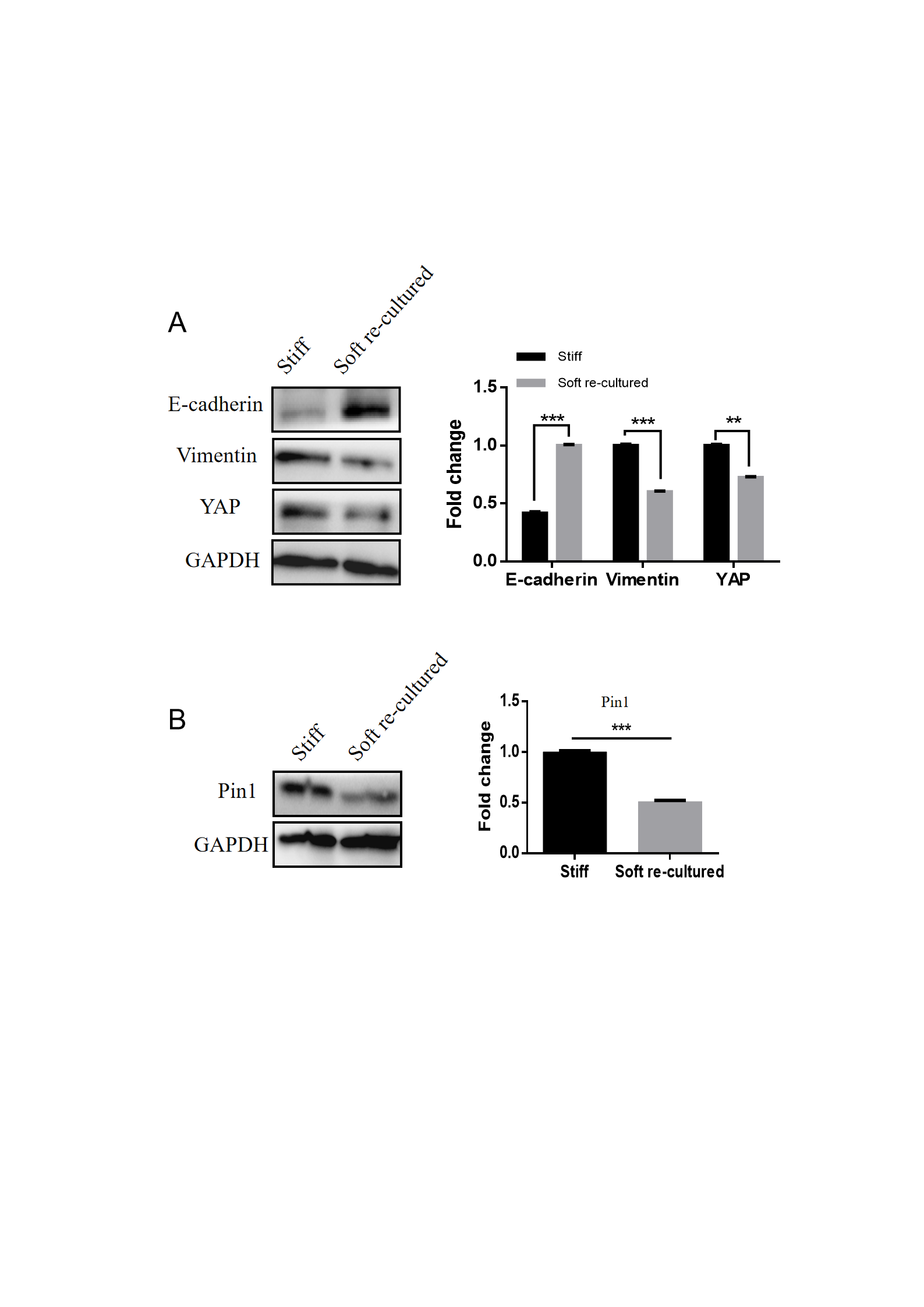


**Supplementary Figure 2.** Western blot analysis of E-cadherin, Vimentin, YAP （A） and Pin1 (B) in hela cell treated with stiff matrix and re-cultured on soft matrix showed expression and quantification, GAPDH was used as a control (***P*<0.01, ****P*<0.001).


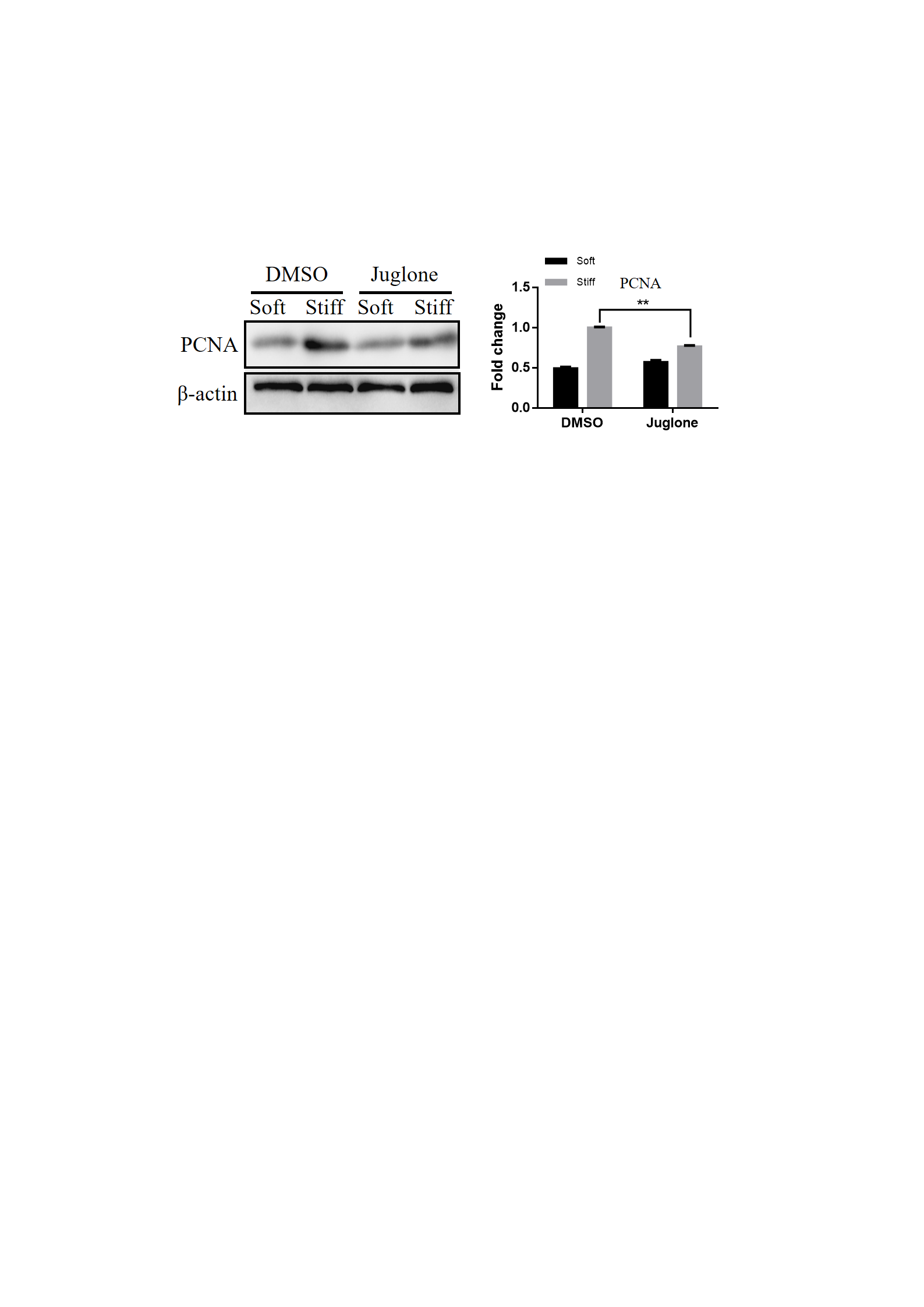


**Supplementary Figure 3**. Western blot analysis of PCNA in hela cell treated with DMSO and Juglone, β-actin was used as a control (***P*<0.01).


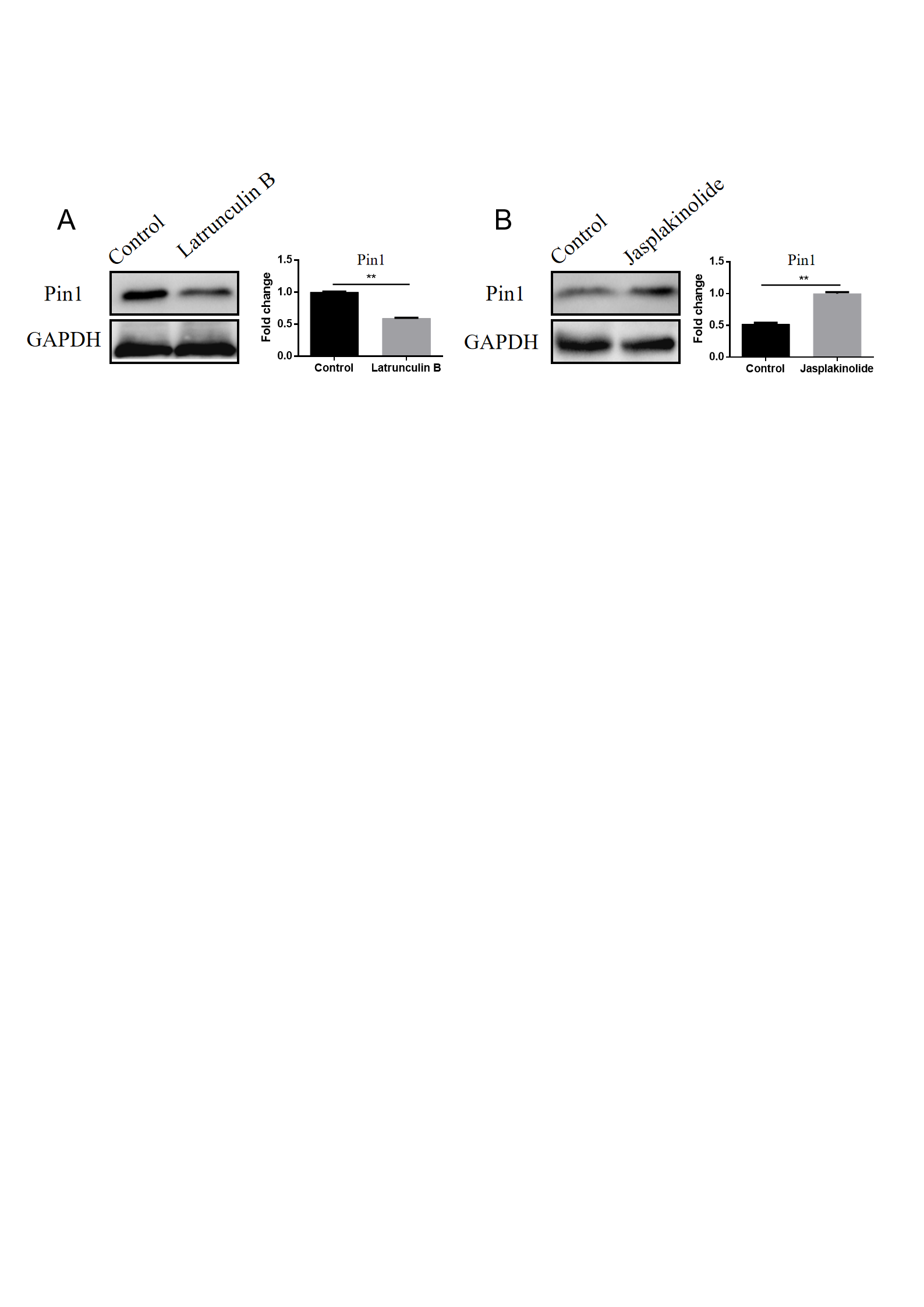


**Supplementary Figure 4.** (**A**) Western blot analysis of Pin1 in Ethanol (Control) and Latrunculin B treating hela cell lysates, GAPDH was used as a control (***P*<0.01). (**B**) Western blot analysis of Pin1 in DMSO (Control) and Jasplakinolide treating hela cell lysates, GAPDH was used as a control (***P*<0.01).
